# Integrated Transcriptomic and Metabolomic Analyses of In Vitro Tuberization of *Siraitia grosvenorii*

**DOI:** 10.3390/plants15213319

**Published:** 2026-10-27

**Authors:** Yuan Xi, Cheng Zeng, Yuqing Su, Qiuling Pang, Mufan Hu, Tailiang Lu, Cuina Fu

**Affiliations:** 1 Guangxi Key Laboratory of Germplasm Innovation and Utilization of Specialty Commercial Crops in North Guangxi, Guangxi Academy of Specialty Crops, Guilin 541004, China13193698720@163.com (Q.P.);; 2 College of Science, China Agricultural University, Beijing 100083, China

**Keywords:** *Siraitia grosvenorii*, in vitro tuberization, metabolome, transcriptome, starch and sucrose metabolism, flavonoid biosynthesis

## Abstract

*Siraitia grosvenorii* (monk fruit) is a cucurbitaceous plant endemic to China that serves as both food and medicine. Its fruit accumulates mogrosides, zero-calorie natural sweeteners approximately 300-fold sweeter than sucrose that are widely used in food and beverages. Whereas tissue-culture studies of *S. grosvenorii* have focused largely on regenerated plantlets, the metabolic events underlying tuber formation in vitro remain poorly characterized. Here, we combined morphological observation, quantification of starch and soluble sugars, time-series transcriptomics, and widely targeted metabolomics to dissect metabolic reprogramming during in vitro tuberization of *S. grosvenorii*. Visible tuber structures emerged at 45 days post-inoculation and accumulated starch continuously throughout development. Starch and sucrose metabolism and flavonoid biosynthesis acted as core pathways regulating the accumulation of carbohydrates and flavonoids. Candidate MYB, bHLH, and ERF transcription factors were screened to explore putative upstream regulation of these metabolic changes. Our results demonstrate that in vitro tuber formation of *S. grosvenorii* is a multi-stage, highly coordinated process, and provide a molecular basis for breeding high-quality propagation materials and for exploiting this species as a source of functional food ingredients and natural sweeteners.

## 1. Introduction

*Siraitia grosvenorii* (Swingle) C. Jeffrey (monk fruit), a perennial vine of the Cucurbitaceae, is a medicinal and edible plant endemic to China [1,2]. The fruit accumulates cucurbitane-type triterpenoid saponins collectively known as mogrosides [3], which are zero-calorie, high-intensity natural sweeteners about 300 times sweeter than sucrose [4,5]. Possessing antioxidant capacity as well as hypoglycemic and hypolipidemic bioactivities, mogrosides can serve as functional food ingredients for alleviating oxidative stress and regulating blood glucose, and represent promising bioactive components for developing functional foods targeting diabetes-related metabolic disorders [6,7]. Apart from bioactive compounds found in its fruit, recent studies have isolated multiple bioactive compounds, including new norcucurbitacin-type triterpenoid glycosides and α-glucosidase inhibitors, from the roots of *S. grosvenorii*, some of which show cytotoxic activity against cancer cell lines [8,9,10,11]. Accordingly, *S. grosvenorii* holds substantial potential for the development of functional foods and natural bioactive products.

In recent years, with the expansion of cultivation areas and the rapid growth of the *S. grosvenorii* industry, disease incidence has risen markedly, and native habitats and wild resources have dwindled [12]. To conserve germplasm resources for sustainable industrial development, tuber induction technology of *S. grosvenorii* is increasingly vital. Tubers not only serve as important nutrient storage organs and propagation materials, but also as effective tools for plant genetic engineering and molecular agriculture [13]. Tubers serve as a material basis for plant overwintering and regrowth in the subsequent year. Their developmental status affects the plant’s growth vigor and tubers with desirable traits can promote plant growth and enhance yield [14,15]. Therefore, understanding the metabolic mechanism of tuber formation helps identify the key factors that improve tuber quality and lays the foundation for improving the yield and quality of *S. grosvenorii* fruits from the source.

In vitro tuber-induction systems have been widely investigated in medicinal and edible tuber crops. In potato (*Solanum tuberosum* L.), the contents of carbohydrates and phenolic bioactive constituents are important indicators reflecting the nutritional quality of tubers [16]. Flavonoid metabolism undergoes substantial remodeling during potato domestication, and such genetic-driven metabolic variations lay the foundation for the diverse nutritional quality of potato tubers [17]. Multiple functional protein groups, including metabolism-related and antioxidant defense proteins, are dynamically regulated throughout tuber induction, development and maturation, underlying the establishment of the tuber storage organ [18]. Anthocyanins represent a major class of flavonoids that contribute to plant pigmentation and defense against diverse environmental stresses [19,20]. Their accumulation in potato is modulated by the light-responsive transcription factor StHY5 [21]. In transgenic potato plants overexpressing StMYB308L, the stem color deepened, and the expression of key flavonoid synthesis genes, including *StCHS*, *StF3′5′H*, *StCHI*, and *StDFR*, was significantly increased [22]. At mechanical injury sites in potato tubers, numerous flavonoid biosynthesis-related genes were significantly upregulated, and the flavonoids exhibited antioxidant and antifungal activities against *Fusarium sulphureum*, the causal agent of potato dry rot, revealing their dual antioxidant and antifungal functions in tubers [23]. The phenylpropanoid pathway provides direct precursors for flavonoid biosynthesis, whereas the flavonoid and lignin branches constitute important downstream pathways of phenylpropanoid metabolism [24]. Exogenous spermidine treatment increased the levels of phenolic acids and lignin monomers, which underwent oxidative crosslinking mediated by POD and H_2_O_2_ to form suberin polyphenolics and lignin that were deposited on the wound surface, establishing a link between phenylpropanoid metabolism and the construction of physical barriers in tubers [25]. And exogenous sugar treatment promotes phenylpropanoid biosynthesis, suggesting that sugar signaling is involved in regulating phenylpropanoid metabolism [26]. Studies involving overexpression of flavonoid biosynthesis regulatory proteins or glucosyltransferases have revealed a competition for carbon flux between flavonoid biosynthesis and starch accumulation, suggesting that the extent of flavonoid glycosylation is more critical than total flavonoid content [27]. Chinese yam (*Dioscorea opposita*) is also an important tuber crop and its microtuber formation is regulated by various plant hormones and tightly coordinated with primary metabolism, including amino acid biosynthesis as well as starch and sucrose metabolism [28]. During tuber development in sweet potato (*Ipomoea batatas* (L.) Lam.), differentially expressed genes were significantly enriched in starch and sucrose metabolism and flavonoid biosynthesis pathways, while differential metabolites exhibited marked changes in these pathways [29].

In tuberous crops, the myeloblastosis (MYB), basic helix-loop-helix (bHLH), and ethylene-responsive factor (ERF) transcription factor families serve as central hubs coordinating organ swelling, carbon partitioning and secondary metabolism. Previous studies on tuberous crops verified that VvMYB61 modulates carbon allocation and flavonoid regulation in storage organs [30]. Tuber-specific StbHLH93 participates in stolon swelling and amyloplast biogenesis in potato [31]. Repressive MYBs (e.g., StMYB3 and StMYBATV) interact with bHLH co-factors to repress anthocyanin biosynthesis during tuber development [32]. Two AP2/ERF transcription factors, StRAP2.7a/b, promote *trans*-zeatin accumulation while repressing *StBRC1A/B* expression, thereby inducing stolon and tuber formation [33].

Findings from these studies on tuberous crops provide valuable references for understanding the metabolic and transcriptional regulation of tuber formation and help us infer regulatory features underlying tuber formation of *S. grosvenorii*. Nevertheless, systematic investigations into the metabolic dynamics during in vitro tuber formation of *S. grosvenorii* remain scarce. Therefore, this study aims to characterize the dynamic changes in transcripts and metabolites during the in vitro tuber development of *S. grosvenorii* and to dissect the regulatory network governing the accumulation of nutritional and functional components. Elucidating the temporal regulatory network underlying the accumulation of nutritional and functional constituents in *S. grosvenorii* in vitro-induced tubers is of great significance for breeding high-quality propagation materials and facilitating the development and utilization of tuber resources as functional food ingredients and natural sweeteners.

## 2. Materials and Methods

### 2.1. Plant Materials

The plant material used in this study was the “Bolin No. 2” cultivar of *S. grosvenorii*, preserved in the Traditional Chinese Medicine Germplasm Resource Nursery of Guangxi Academy of Specialty Crops. Explants were collected from healthy mother plants and inoculated onto the MS (Cat. No. M8521; Beijing Solarbio Science & Technology Co., Ltd., Beijing, China) medium containing 0.1 mg/L 6-benzylaminopurine (6-BA, Cat. No. B802626; Shanghai Macklin Biochemical Co., Ltd., Shanghai, China), 0.01 mg/L naphthaleneacetic acid (NAA, Cat. No. N814716; Shanghai Macklin Biochemical Co., Ltd., Shanghai, China)), 30 g/L sucrose (Cat. No. S8270; Beijing Solarbio Science & Technology Co., Ltd., Beijing, China, and 7 g/L agar (bacteriological grade; Chembase Biotechnology, Beijing, China) (pH5.8) and cultured to obtain aseptic seedlings.

When the aseptic seedlings reached 8–10 cm in length, stem segments with axillary buds were excised under sterile conditions to serve as explants for tuber induction and cultured on 1/2 MS medium supplemented with 0.01 mg/L NAA, 0.50 mg/L 6-BA, 1.00 mg/L paclobutrazol (PP333, Cat. No. P109932; Aladdin Biochemical Technology Co., Ltd., Shanghai, China), 60 g/L sucrose and 7 g/L agar (pH5.8). Cultures were incubated in a tissue-culture room at 22 °C with a 10 h/d photoperiod and 1500 lx light intensity. The day of inoculation was defined as 0 d, with 10 explants inoculated per culture flask. Twelve culture flasks were randomly selected, consisting of three biological replicates with four flasks per replicate. Stem segments (1–2 cm) containing axillary buds were excised from uniformly growing explants and tuber-bearing materials were collected once tubers formed at later culture stages as samples. Sampling was carried out in a laminar flow cabinet every 15 d until 90 d post-inoculation. Collected samples were rinsed with RNase-free water, blotted dry with sterile filter paper, placed into 5 mL sterile centrifuge tubes, snap-frozen in liquid nitrogen for 10 min, and stored at −80 °C for subsequent analyses. For brevity, these time points are abbreviated as 0 d, 15 d, 30 d, 45 d, 60 d, 75 d and 90 d in the following results, figure legends and graphs.

### 2.2. Determination of Fructose, Sucrose, Glucose and Starch Contents

The contents of fructose, sucrose, glucose and starch were determined using commercial 96-well microplate assay kits from AIDISHENG (Yancheng, China). The catalog numbers were ADS-W-TDX071 for fructose, ADS-W-TDX040-100 for glucose, and ADS-W-DF016 for sucrose and ADS-W-DF001 for starch. All measurements were performed strictly following the manufacturer’s instructions, with three biological replicates for each assay. Statistical analysis was performed in GraphPad Prism 6 software (GraphPad Software, San Diego, CA, USA). Data were presented as mean ± SEM (*n* = 3 biological replicates). One-way analysis of variance (ANOVA) was applied to assess differences among groups across different time points. The homogeneity of variances was verified using the Brown–Forsythe test. Tukey’s HSD multiple pairwise comparisons were conducted for all pairwise combinations. Statistical significance was defined at *p* < 0.05. * *p* < 0.05, ** *p* < 0.01, *** *p* < 0.001, **** *p* < 0.0001, and ns indicates no significant difference. Full Tukey comparison results (mean difference, 95% confidence interval and significance summary for all pairwise comparisons) are provided in Supplementary Table S1.

### 2.3. Quantitative Real-Time PCR Analysis

Quantitative real-time PCR (qRT-PCR) was performed for gene expression validation. Total RNA used in this assay was extracted by Metware Biotechnology Co., Ltd. (Wuhan, China), which was identical to the RNA used for transcriptome sequencing. RNA samples received from the company were pooled at the same time point from three replicates, and four technical replicate reactions were run for each pooled sample to minimize technical variation. First-strand cDNA was synthesized from 1 µg of total RNA using the HiScript II 1st Strand cDNA Synthesis Kit (Vazyme, Nanjing, China, Cat. No. R223-01). qRT-PCR was performed on a Bio-Rad CFX96 Real-Time PCR Detection System (Bio-Rad Laboratories, Hercules, CA, USA) using ChamQ Universal SYBR qPCR Master Mix (Without ROX) (Vazyme, Nanjing, China, Cat. No. Q321-02). The thermal cycling conditions were: 95 °C for 30 s, followed by 40 cycles of 95 °C for 10 s and 60 °C for 30 s. A melt curve analysis was performed at the end of each run to verify primer specificity. UBQ (Ubiquitin) served as the internal reference gene. Relative expression was calculated using the 2^−ΔΔCq^ method, with the 0 d sample as the calibrator. Statistical significance was determined by independent-samples Welch’s *t*-test on ΔCq values, with Benjamini–Hochberg false discovery rate (FDR) correction applied across all 36 genes–time-point comparisons. Primers used in this study can be found in Supplementary Table S2.

### 2.4. Transcriptome Analysis

RNA-seq was conducted by Metware Biotechnology Co., Ltd. (Wuhan, China). Raw sequencing reads were filtered to remove low-quality sequences (with Q ≤ 20) and reads with more than 10% N sequences using fastp software (version 0.23.2) [34]. Clean reads were mapped to the *S. grosvenorii* reference genome [1] via HISAT2 [35] (https://gigadb.org/dataset/100452, accessed on 16 August 2025). Read counts for each gene were calculated by featureCounts (version 2.0.3) [36], and gene expression levels were normalized to FPKM values. Differentially expressed genes (DEGs) were screened using DESeq2 (version 1.22.1) [37,38] with the thresholds of ∣log_2_FC∣ > 1 and adjusted false discovery rate (FDR) < 0.05. Plant transcription factors were predicted based on three databases including iTAK [39], PlnTFDB [40] and PlantTFDB [41]. Functional annotation of DEGs was performed against the Swiss-Prot database. Pathway enrichment analysis was implemented based on the Kyoto Encyclopedia of Genes and Genomes (KEGG) database [42] (http://www.genome.jp/kegg/, accessed on 18 August 2025). Weighted gene co-expression network analysis (WGCNA) was performed using the WGCNA R package based on normalized gene expression values (FPKM). The pickSoftThreshold function was applied to identify the optimal soft-thresholding power, with RsquaredCut set to 0.85 to meet the scale-free topology criterion. The soft-thresholding power was finally set to 15 to achieve scale-free topology. Modules were identified with default parameters. Module membership (MM, also known as kME) represents the correlation between each gene and the module eigengene.

### 2.5. Metabolome Analysis

Metabolomic extraction and analysis were conducted by Metware Biotechnology Co., Ltd. (Wuhan, China). Samples were vacuum freeze-dried and ground into powder. Next, 50 mg of sample powder was mixed with 1200 μL of pre-cooled (−20 °C) 70% aqueous methanol containing an internal standard and vortexed once every 30 min for 30 s, six times in total. After centrifugation at 12,000 rpm for 3 min, the supernatant was filtered through a microporous membrane (0.22 μm pore size) and stored in the injection vial for Ultra-Performance Liquid Chromatography-Tandem Mass Spectrometry (UPLC-MS/MS) analysis. Three biological replicates were prepared for each sampling time point. Metabolite profiling was performed on a UPLC-ESI-MS/MS system (UPLC, ExionLC™AD, SCIEX, Framingham, MA, USA) [43]. Separation was conducted on an Agilent SB-C18 (1.8 µm, 2.1 mm × 100 mm) with mobile phase A (0.1% formic acid in water) and solvent B (0.1% formic acid in acetonitrile). The gradient elution program was as follows: initial 95% A/5% B, a linear gradient to 5% A/95% B within 9 min (held for 1 min), then returned to the initial ratio within 1.1 min and maintained for 2.9 min. The flow rate was 0.35 mL/min, the column temperature was 40 °C, and the injection volume was 2 μL. The ESI source parameters were as follows: source temperature 500 °C; ion spray voltage (IS) 5500 V (positive ion mode)/−4500 V (negative ion mode); ion source gas I (GSI), gas II (GSII), and curtain gas (CUR) were set at 50, 60, and 25 psi, respectively; the collision-activated dissociation (CAD) was high. MRM mode was used for metabolite quantification, with declustering potential (DP) and collision energy (CE) optimized for each transition. Metabolite identification was performed by matching retention time, accurate mass, and fragment spectra against the in-house Metware metabolite database.

All statistical analyses were performed using R software (version 4.1.2) [44]. Unit-variance scaling (UV scaling, also known as auto-scaling or Z-score normalization) was applied for principal component analysis (PCA), hierarchical clustering heatmap and K-means clustering. Differential metabolites were screened based on VIP > 1 (from the OPLS-DA, MetaboAnalystR package) and |Log_2_FC| ≥ 1, and permutation testing with 200 permutations was applied to assess model overfitting. Cumulative R^2^X, R^2^Y and Q^2^ values of the model and permutation test results with 200 iterations are summarized in the Supplementary Table S3. Identified metabolites were annotated via the KEGG Pathway database for functional analysis [45] (http://www.kegg.jp/kegg/pathway.html, accessed on 18 August 2025).

## 3. Results

### 3.1. Morphological Development and Dynamic Changes of Starch and Soluble Sugars During In Vitro-Induced Tuberization of S. grosvenorii

**Figure 1:**
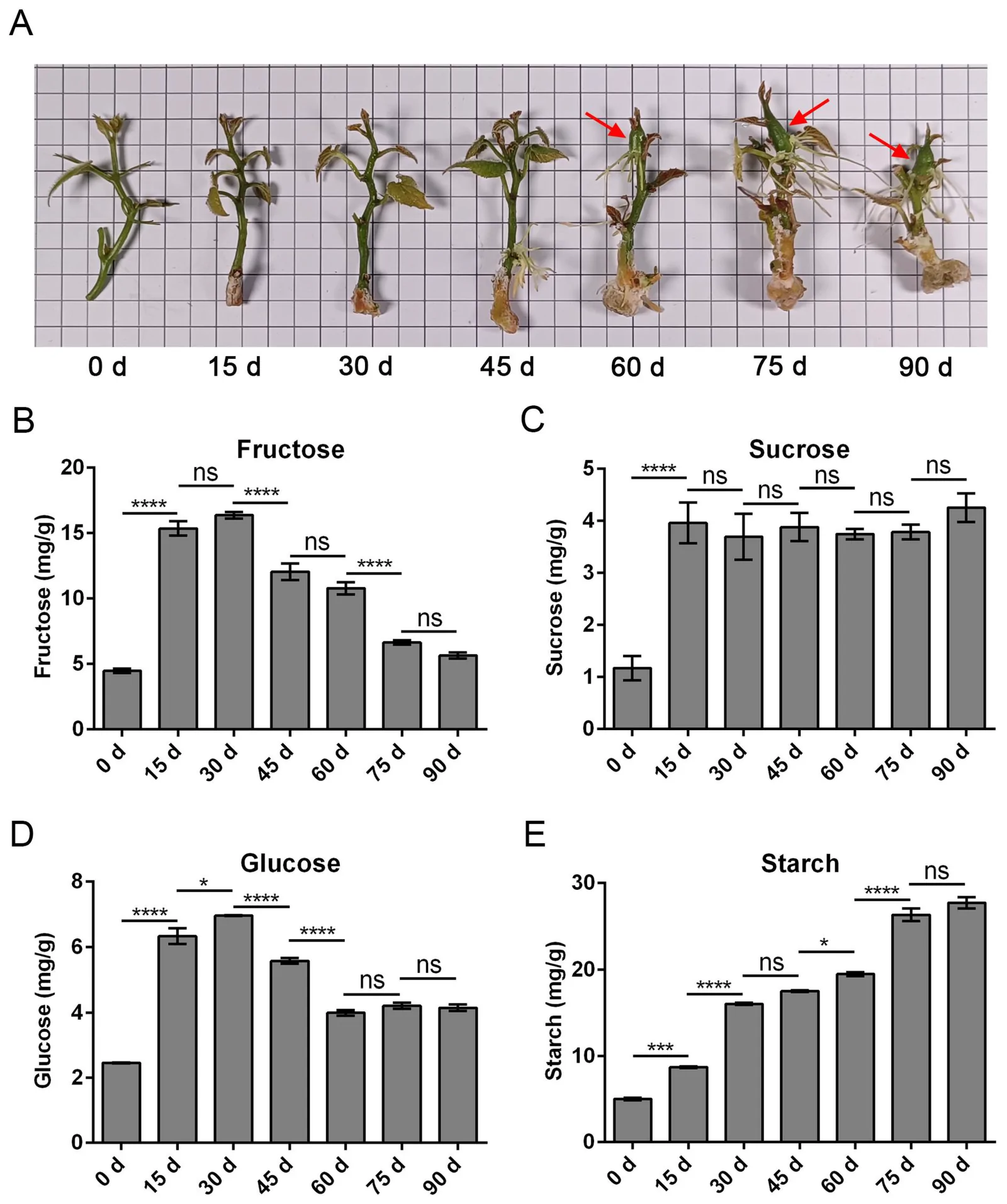
(**A**) Morphological changes: red arrows indicate expanded tuber; each grid square represents 1 cm in side length. (**B**) Fructose content. (**C**) Sucrose content. (**D**) Glucose content. (**E**) Starch content. Data are presented as mean ± SEM, *n* = 3 biological replicates. One-way ANOVA revealed significant differences in carbohydrate contents across different culture days, followed by Tukey’s HSD multiple pairwise comparisons. * *p* < 0.05, *** *p* < 0.001, **** *p* < 0.0001, and ns indicates no significant difference at *p* ≥ 0.05. Comparisons were shown between adjacent time points.

To elucidate the temporal dynamics of carbohydrate metabolism linked to quality formation during in vitro-induced tuberization of *S. grosvenorii*, we continuously monitored the morphological changes over a 0–90 day culture period and quantified the dynamic contents of fructose, glucose, sucrose and starch. Samples were harvested on days 0, 15, 30, 45, 60, 75, and 90 after inoculation (Figure 1A). From 0 d to 30 d, the explants exhibited continuous vegetative growth, with slight slow-proliferating callus formed at the basal region, and no tuber structures observed. From 45 d to 60 d, tuberous structures appeared and gradually underwent swelling; meanwhile, aerial roots formed on some cultures. Tubers underwent continuous expansion from 75 d to 90 d. We simultaneously determined the contents of fructose, sucrose, glucose and starch. Fructose content increased continuously from 0 d to 30 d, peaked at 30 d, and then declined steadily to 90 d (Figure 1B). Glucose exhibited a similar trend to fructose, reaching its maximum concentration at 30 d and decreasing gradually thereafter (Figure 1D). Sucrose content increased rapidly from 0 d to 15 d and remained at a relatively stable high level from 15 d to 75 d, with a slight maximum at 90 d (Figure 1C). Starch content increased continuously throughout the culture period, with the minimum observed at 0 d and the maximum at 90 d (Figure 1E).

### 3.2. Metabolomic Profiling During In Vitro-Induced Tuberization of S. grosvenorii

**Figure 2:**
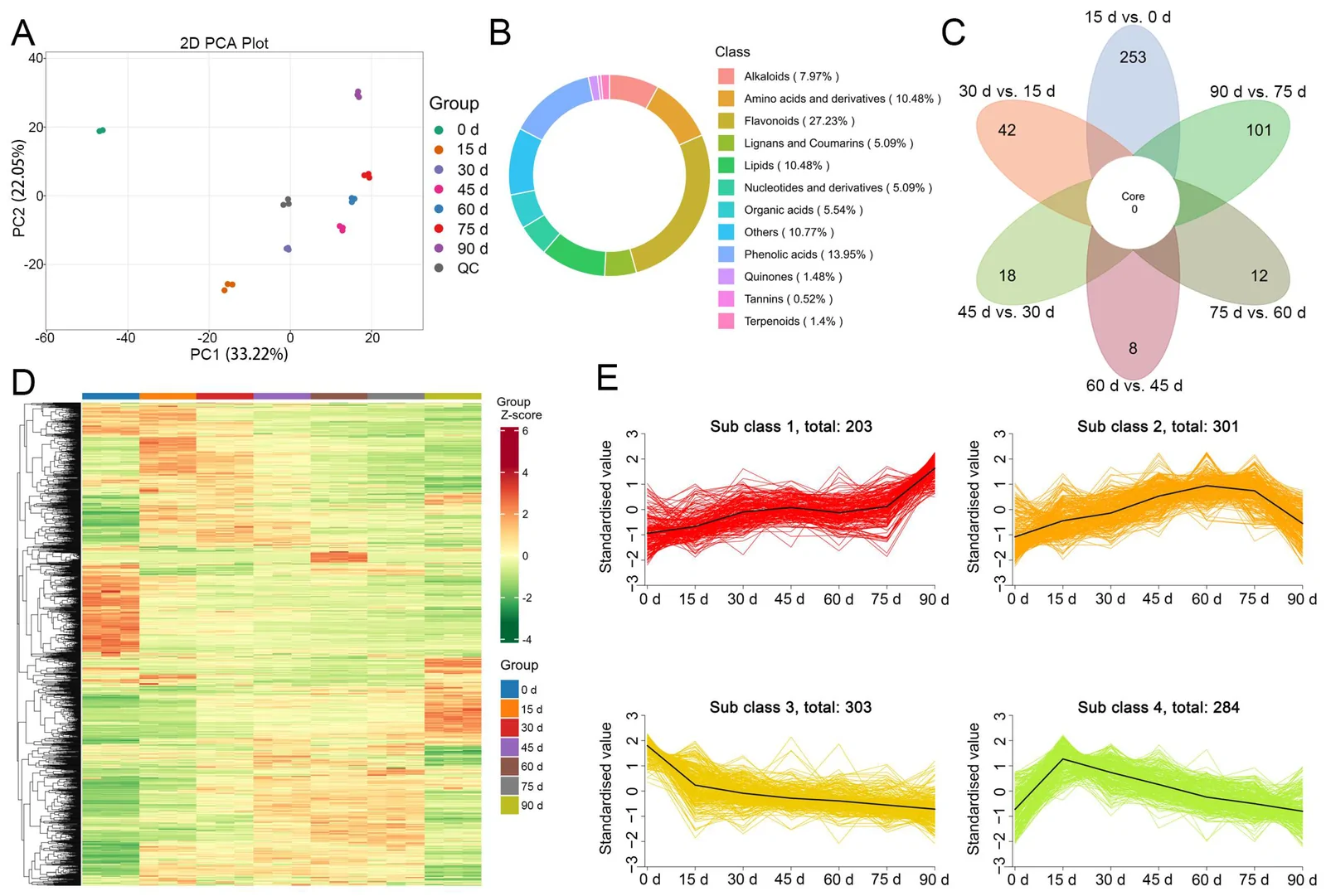
(**A**) Principal component analysis (PCA). (**B**) Donut chart of different categories of metabolites. (**C**) Venn diagram of DAMs among groups. (**D**) Hierarchical clustering heatmap of DAMs. (**E**) K-Means clustering of DAMs. Each line represents one DAM, and the black line indicates the average expression trend of each subclass.

A total of 1355 metabolites were detected and identified from tuber samples collected at seven time points (0, 15, 30, 45, 60, 75, 90 d) using UPLC-MS/MS. Principal component analysis (PCA) results showed clear separation among stages, with tight clustering of biological replicates within each stage, indicating high reproducibility (Figure 2A). Notably, the 0 d samples were the most distinct from subsequent time points. Differentially accumulated metabolites (DAMs) were annotated and subsequently classified into 12 distinct groups (Figure 2B). Flavonoids constituted the largest portion at 27.23%, followed by phenolic acids at 13.95%, unclassified metabolites (others) at 10.77%, lipids at 10.48%, amino acids and derivatives at 10.48%, alkaloids at 7.97%, organic acids at 5.54%, nucleotides and derivatives at 5.09%, lignans and coumarins at 5.09%, quinones at 1.48%, terpenoids at 1.4%, and tannins at the lowest proportion of 0.52%. The Venn diagram showed that the metabolic responses were highly stage-specific (Figure 2C); the 15 d vs. 0 d comparison contained the most unique DAMs, followed by 90 d vs. 75 d and 30 d vs. 15 d. The hierarchical clustering heatmap exhibited that a large proportion of metabolites were abundant in the 0 d and 15 d groups and continuously downregulated from 30 d to 75 d, whereas another subset of metabolites was gradually upregulated with the culture time (Figure 2D). K-means clustering divided the DAMs into four clusters (Figure 2E). Class 1 metabolites continuously increased throughout the development stages. Class 2 metabolites exhibited an upward trend and began to decline after 60 d. Class 3 metabolites consistently decreased, with a pronounced decline from 0 d to 15 d. Class 4 metabolites increased rapidly only from 0 d to 15 d and then declined continuously. Collectively, these metabolome analyses revealed remarkable time-dependent metabolic shifts throughout tuber formation in *S. grosvenorii*.

### 3.3. Transcriptomic Profiling During In Vitro-Induced Tuberization of S. grosvenorii

**Figure 3:**
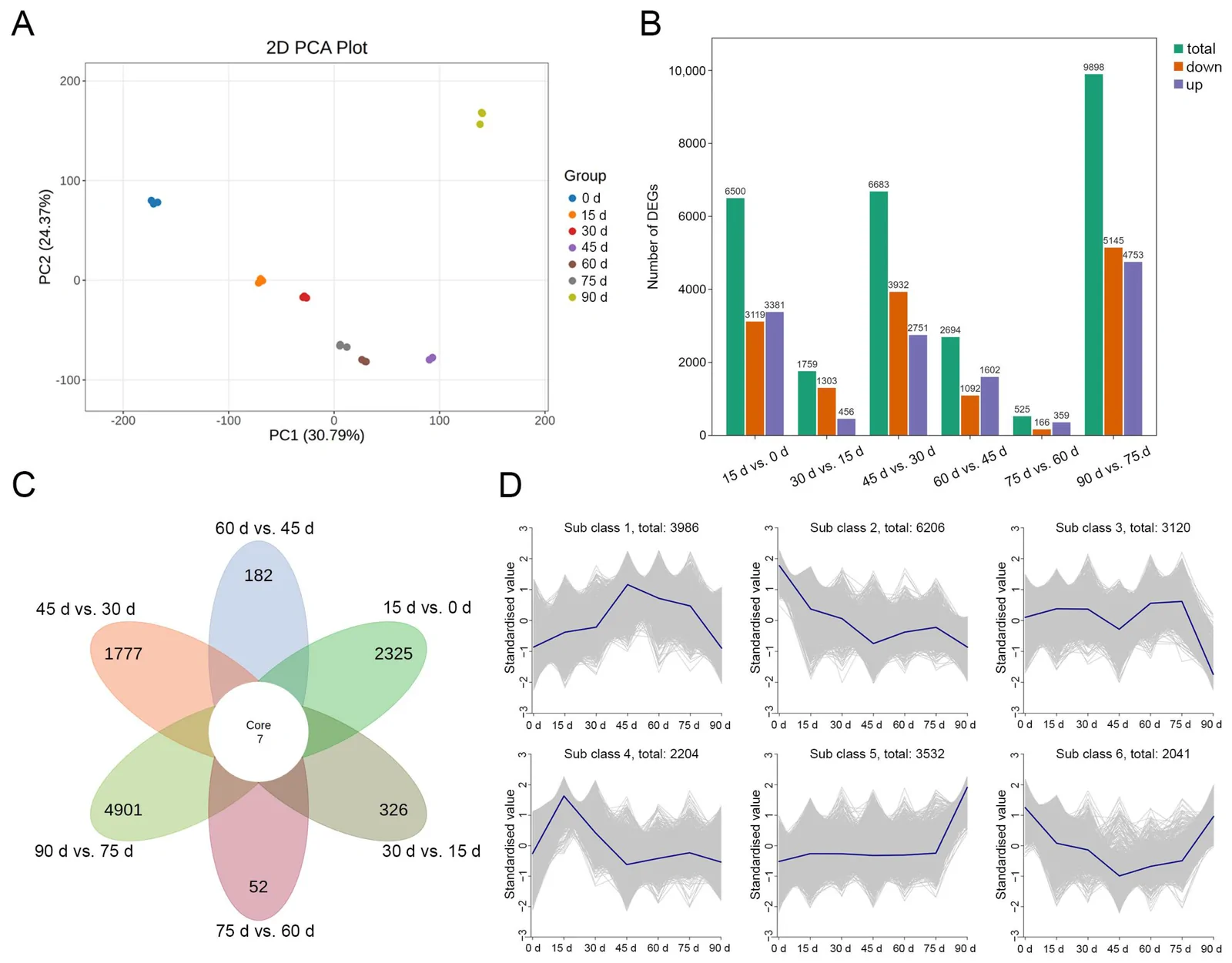
(**A**) PCA of all differentially expressed genes (DEGs). (**B**) Bar chart of DEGs. (**C**) Venn diagram showing shared and unique DEGs across six pairwise comparison groups. (**D**) K-means clustering analysis of DEGs based on standardized temporal expression values across seven culture stages. Each line represents one DEG, and the blue line indicates the average expression trend of each subclass.

To further decipher the transcriptional regulatory mechanisms underlying metabolic reprogramming, we performed transcriptome sequencing. PCA results showed that biological replicates within each group clustered tightly and groups were distinctly separated, indicating good reproducibility and remarkable transcriptional divergence (Figure 3A). The bar chart of differentially expressed genes (DEGs) revealed that three comparison groups exhibited the largest number of DEGs, namely 90 d vs. 75 d, 45 d vs. 30 d and 15 d vs. 0 d among all six pairwise comparisons (Figure 3B). The Venn diagram showed that seven core DEGs were shared across all comparison groups, whereas most DEGs were stage-specific (Figure 3C). The 90 d vs. 75 d comparison possessed the largest number of unique DEGs (4901), followed by 15 d vs. 0 d (2325) and 45 d vs. 30 d (1777). K-means clustering classified all DEGs into six distinct sub-clusters based on their temporal expression patterns (Figure 3D). Each sub-cluster exhibited unique expression trends across the seven culture stages, indicating stage-specific transcriptional regulation. Class 2 contained the largest number of DEGs (6206), whereas class 6 had the fewest (2041). Genes in classes 2 and 6 were highly expressed at 0 d and then declined in the early culture period. In contrast, class 4 genes peaked at 15 d, class 1 reached the highest expression at 45 d, and class 5 was continuously upregulated at the late culture stage. Overall, transcriptomic profiling indicated that DEGs exhibited varied expression patterns during in vitro tuberization of *S. grosvenorii*.

Combined with phenotypic and multi-omics data, we further explored the starch and sucrose metabolism and flavonoid biosynthesis pathways, which have been widely reported to regulate tuber formation in multiple tuberous crop species, as described in our introduction.

### 3.4. Temporal Dynamics of Starch and Sucrose Metabolism Pathways During In Vitro-Induced Tuberization of S. grosvenorii

**Figure 4:**
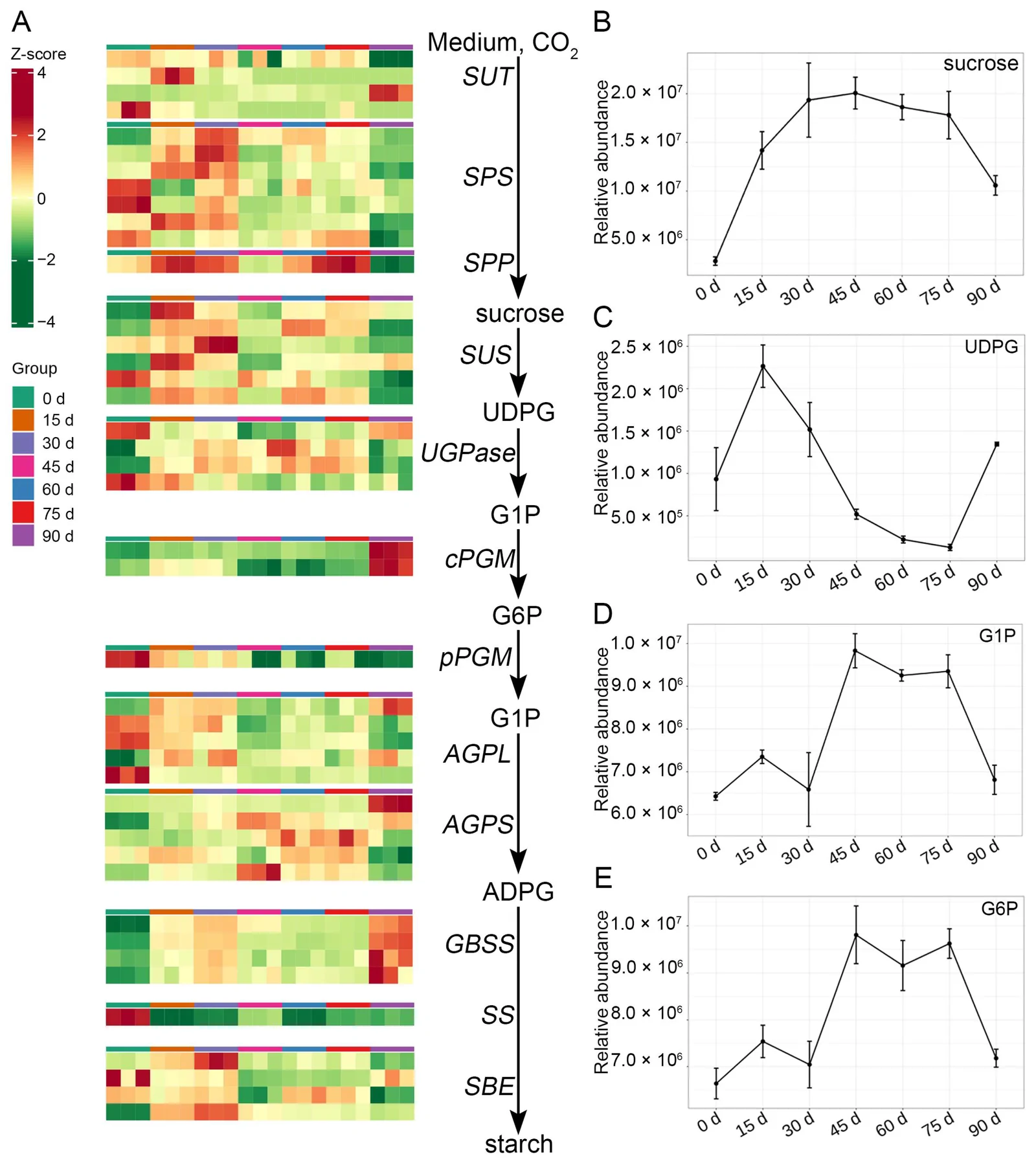
(**A**) The heatmaps of DEGs involved in the starch and sucrose metabolism pathway during 0–90 d. (**B**–**E**) Line charts showing changes in the relative abundance of related metabolites over culture stages.

Based on the above analysis, we further characterized the temporal expression patterns of core genes and the dynamic accumulation of corresponding metabolites involved in starch and sucrose metabolism across all stages. Heatmap analysis revealed divergent temporal expression patterns of *S. grosvenorii* DEGs annotated as participants in sucrose and starch metabolism, including sucrose transport protein (*SUT*), sucrose phosphate synthase (*SPS*), sucrose phosphate phosphatase (*SPP*), sucrose synthase (*SUS*), UTP-glucose-1-phosphate uridylyltransferase (*UGPase*), phosphoglucomutase (*PGM*), ADP-glucose pyrophosphorylase small subunit (*AGPS*), ADP-glucose pyrophosphorylase large subunit (*AGPL*), granule-bound starch synthase (*GBSS*), starch synthase (*SS*) and starch branching enzyme (*SBE*) (Figure 4A). The *SUTs* were upregulated and the corresponding proteins transported sucrose from the medium into the tissues. Most copies of *SPS*, *SPP* and *SUS* genes were upregulated during the early culture stage (15–30 d) and late culture stage (60 d–75 d), consistent with the relatively high sucrose content from 30 to 75 d and its decline from 75 to 90 d (Figure 4B). The *UGPase* genes showed different expression patterns across the developmental stages, accompanied by fluctuating UDP-glucose (UDPG) levels, suggesting dynamic interconversion between soluble sugars and starch (Figure 4C). The expression of cytosolic *PGM* (*cPGM*) and plastidic *PGM* (*pPGM*) genes indicated their roles in glucose-1-phosphate (G1P) and glucose-6-phosphate (G6P) interconversion during sucrose-starch transition (Figure 4D,E). Genes involved in starch biosynthesis exhibited distinct temporal expression patterns. The *AGPL* and *AGPS* genes showed asynchronous expression, with some members highly expressed at early stages, whereas others were strongly upregulated at later time points. *GBSS* was markedly induced at 90 d. *SS* displayed high expression at 0 d, followed by reduced transcript levels. The *SBE* showed divergent expression trajectories, with distinct subgroups preferentially expressed in early, middle, and late stages, respectively. The coordinated shifts in related genes and intermediate metabolites indicated that sucrose and starch metabolism was activated, consistent with the accumulation of sucrose and starch during in vitro tuber formation of *S. grosvenorii*.

### 3.5. Temporal Dynamics of Flavonoid Biosynthesis During In Vitro-Induced Tuberization of S. grosvenorii

**Figure 5:**
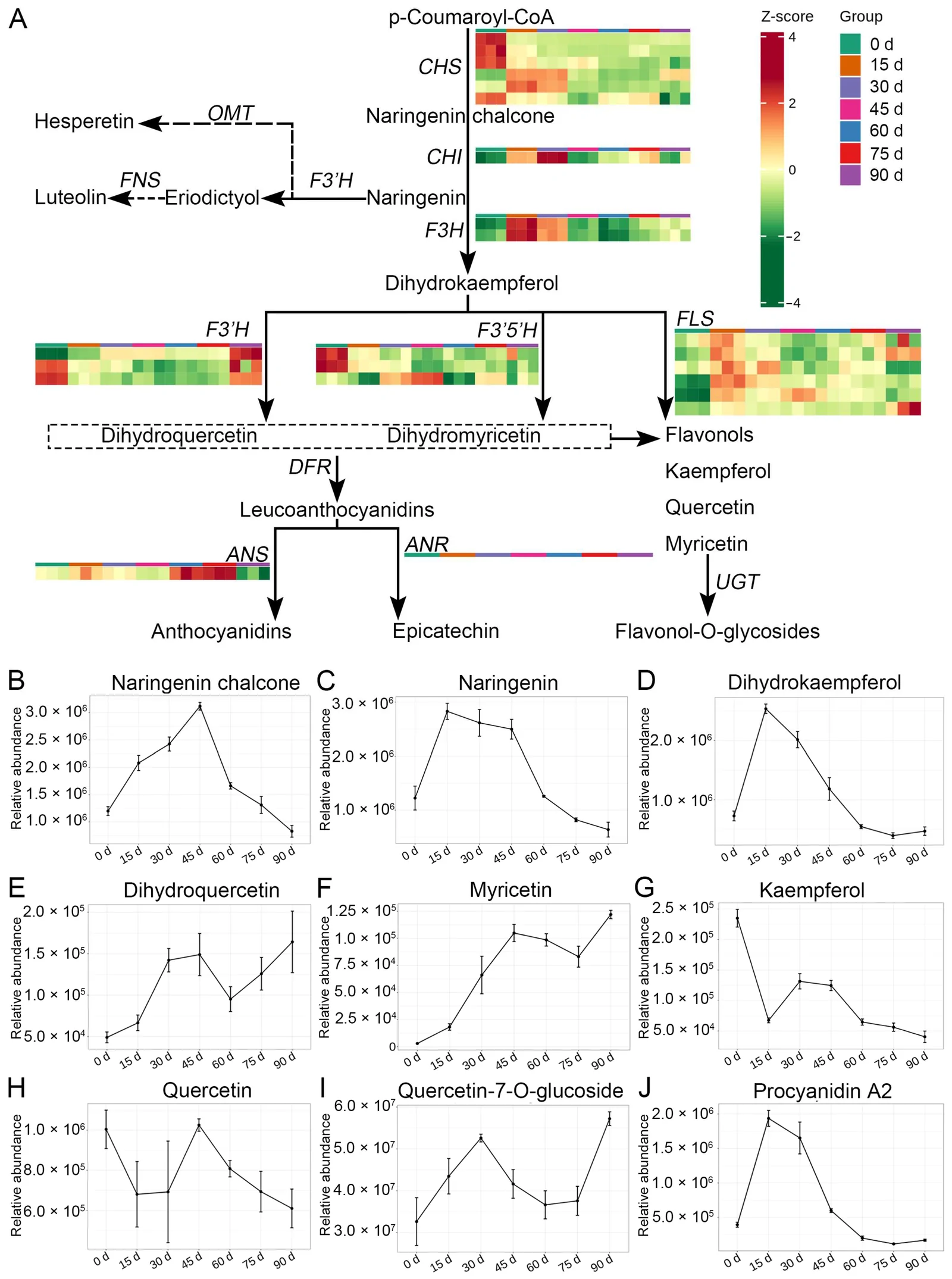
(**A**) The heatmaps of DEGs involved in the flavonoid biosynthesis pathway during 0–90 d. (**B**–**J**) Line charts showing changes in the relative abundance of flavonoids over culture stages.

Beyond primary carbon metabolism represented by starch and sucrose metabolism, flavonoids constitute important bioactive secondary metabolites in *S. grosvenorii*. We further characterized the changes in DEGs and DAMs involved in the flavonoid biosynthesis pathway by integrating transcriptome and widely targeted metabolome datasets. Heatmap analysis revealed divergent temporal expression patterns of core genes including chalcone synthase (*CHS*), chalcone isomerase (*CHI*), flavanone 3-hydroxylase (*F3H*), flavonoid 3′-hydroxylase (*F3′H*), flavonoid 3′,5′-hydroxylase (*F3′5′H*), flavonol synthase (*FLS*), leucoanthocyanidin dioxygenase (*LDOX*/*ANS*) and anthocyanidin reductase (*ANR*) (Figure 5A). Most upstream backbone biosynthetic genes (*CHS*, *CHI*, *F3H*, *FLS*) were markedly upregulated during the early culture stage (0 d–30 d) to drive the formation of flavonoid skeletons (Figure 5). *F3′H* and *F3′5′H* were highly expressed at 0 d; their transcripts declined during the middle period yet remained at a certain level. Notably, *F3′H* showed reactivation at 90 d. *FLS* members were actively expressed from 15 d to 30 d and showed a slight rebound at 90 d. ANS and ANR exhibited distinct expression patterns. The line charts showed flavonoid metabolites including naringenin chalcone, naringenin, dihydrokaempferol, dihydroquercetin, myricetin, quercetin, kaempferol, quercetin-7-O-glucoside and procyanidin A2 (Figure 5B,J). Naringenin chalcone, naringenin, and dihydrokaempferol all showed elevated accumulation during the middle developmental stage, yet with shifted peak timing: naringenin chalcone reached its maximum abundance at 45 d, whereas naringenin and dihydrokaempferol peaked at 15 d. Following their respective peaks, the relative abundances of all three metabolites gradually decreased and remained at low levels in later stages. Dihydroquercetin, myricetin and quercetin-7-O-glucoside displayed an overall increasing trend throughout development, with a transient minor dip at 60 d and 75 d. Kaempferol showed a general decreasing tendency, with only transient increases at 30 d and 45 d. Quercetin exhibited a dynamic pattern of initial decrease, followed by a rise and a subsequent decline. Procyanidin A2 reached its accumulation peak at 15 d and decreased continuously thereafter. The flavonoid biosynthetic pathway was globally activated during tuber development. Metabolites involved in flavonoid skeleton biosynthesis accumulated markedly at the early-to-middle stage to supply substrates for downstream branching pathways. Flavonoid derivatives modified by hydroxylation and glycosylation were upregulated at the middle and late developmental stages. Nevertheless, metabolic flux toward the anthocyanin branch was relatively limited. Procyanidin A2 decreased substantially after reaching its mid-stage peak, suggesting that flavonoid metabolic flux was preferentially allocated to the flavonol branch.

### 3.6. MYB, bHLH and ERF Transcription Factors Associated with In Vitro-Induced Tuberization of S. grosvenorii

**Figure 6:**
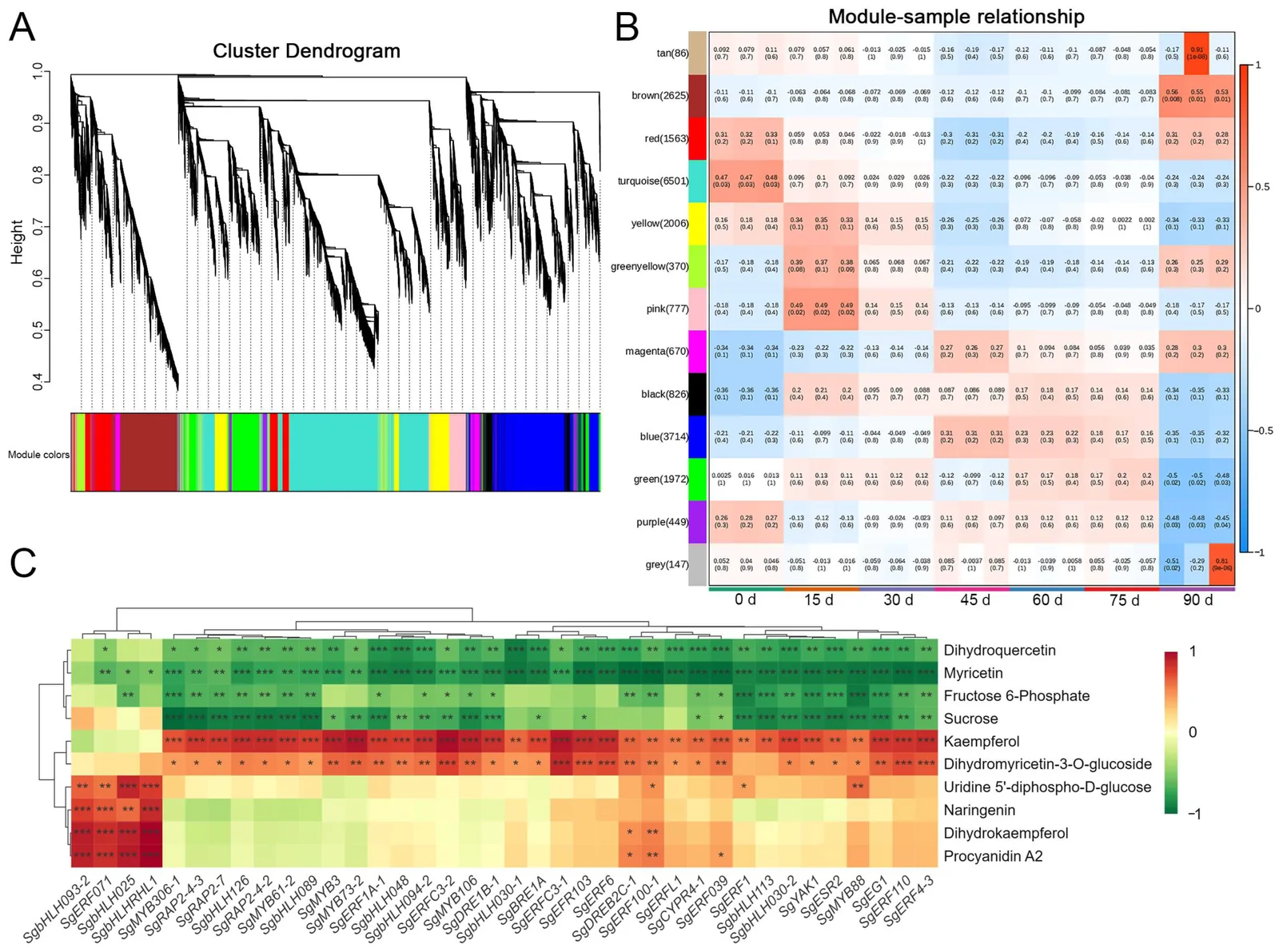
(**A**) WGCNA. (**B**) Module-sample relationship heatmap. (**C**) Pearson correlation heatmap between candidate transcription factors and key metabolites of starch and sucrose metabolism and flavonoid biosynthesis pathways. Rows represent metabolites; columns represent candidate transcription factors. Red and green represent positive and negative correlation coefficients, respectively. Correlations with absolute values of the Pearson correlation coefficient > 0.8 are displayed. * *p* < 0.05, ** *p* < 0.01, *** *p* < 0.001.

To identify core transcription factors (TFs) participating in the regulation of starch and sucrose metabolism and flavonoid biosynthesis pathways during tuber development, we generated a WGCNA dendrogram, which illustrated the co-expression relationships among genes and exhibited clear boundaries among modules, indicating reliable module partitioning (Figure 6A). Modules brown, turquoise and pink were further selected based on the module-sample relationship heatmap (Figure 6B). These three modules showed significant positive correlations at the early, early-middle and late tuber developmental stages, respectively. This pattern matched the sequential high-expression features of structural genes involved in starch and sucrose metabolism and flavonoid biosynthesis across developmental phases. These modules were therefore selected for subsequent analyses. TFs of MYB, bHLH and ERF with module membership (MM) > 0.8 were screened. These transcription factors, together with representative metabolites involved in starch and sucrose metabolism and flavonoid biosynthesis, were used to construct a gene-metabolite co-expression network to decipher the putative relationships between these transcription factors and target metabolites (Figure 6C). Highly divergent correlation patterns were observed among these TFs. A set of TFs on the left side of the heatmap, such as *SgbHLH093-2*, exhibited strong significant positive correlations with uridine 5′-diphospho-D-glucose, dihydrokaempferol, naringenin and procyanidin A2. Most TFs were significantly positively correlated with kaempferol and dihydromyricetin-3-O-glucoside, whereas they exhibited negative correlations with dihydroquercetin, myricetin, sucrose and fructose-6-phosphate. Together, this co-expression network revealed putative associations between these transcription factors and metabolites related to starch and sucrose metabolism and flavonoid biosynthesis pathways.

## 4. Discussion

In the present study, integrative transcriptome and widely targeted metabolome analyses were performed across seven developmental time-points (0–90 d) to preliminarily uncover the dynamic changes in starch and sucrose metabolism, flavonoid biosynthesis, as well as the regulatory networks of candidate transcription factors, during in vitro tuberization of *S. grosvenorii*, providing valuable insights for the exploitation and utilization of *S. grosvenorii* as a source of natural sweeteners and functional food ingredients.

A limitation of the present study is the absence of a non-tuber-forming control group. All explants were cultivated on the same tuber-inducing medium. Accordingly, the time-dependent transcriptomic and metabolic shifts detected in this work cannot be fully discriminated as tuber-specific regulatory events from general physiological responses triggered by prolonged in vitro culture, high exogenous sucrose or progressive tissue senescence. While our phenotypic data show obvious tuber formation along the time series, we cannot exclude that part of the observed molecular alterations are common stress or aging responses independent of tuber organogenesis. Future experiments with a non-tuber induction control will help separate these two categories of molecular events and further validate the regulatory network underlying tuber development.

Combined transcriptomic and metabolomic data revealed that during the early-to-middle stage of tuber formation (0–30 d), most genes involved in starch and sucrose metabolism and flavonoid biosynthesis were synchronously and significantly upregulated. This indicates that tuber formation depends on basic carbon metabolism to sustain rapid cell proliferation and substantial energy demands, and massive biosynthesis of antioxidant flavonoids may help counteract in vitro-culture-associated stresses including oxidative stress and mechanical damage [46,47]. The rapid increase in sucrose content, supported by both physiological measurements and metabolomic data, may derive from two sources: exogenous sucrose supplied in the medium and endogenous biosynthesis within in vitro-induced tubers. However, the relative contributions of absorbed exogenous sucrose and de novo-synthesized sucrose cannot be distinguished at present, and stable isotope labeling experiments would be required to resolve this question. In starch and sucrose metabolism and flavonoid biosynthesis, some intermediate metabolites such as ADP-glucose (ADPG) and dihydromyricetin were not detected in the widely targeted metabolomics data. This may be attributed to the rapid metabolic flux of the pathway, in which intermediates are quickly shunted to downstream reactions. Alternatively, the failure to detect these compounds may result from the limitations of widely targeted metabolomics. At the late culture stage, AGPase acts as the rate-limiting enzyme for starch biosynthesis in sink organs, forming a functional heterotetramer composed of two large regulatory subunits (AGPL) and two small catalytic subunits (AGPS) [48]. Studies have demonstrated that the holoenzyme activity of AGPase is not entirely determined by transcript levels and requires post-translational modification for activation [49]. Our transcriptome data revealed divergent expression patterns of *AGPL* and *AGPS* during tuber development. Nevertheless, co-expression of both subunits remains the foundation for normal starch biosynthesis. In addition, the expression patterns of these two pathways exhibited distinct divergence: some key starch biosynthesis genes centered on the conversion of sucrose to starch, such as *AGPS* and *GBSS*, maintained high expression levels. The expression of genes responsible for the core skeleton formation of flavonoids, including *CHS* and *F3H*, gradually declined. In root and tuber crops, enhanced flux toward phenylpropanoid-derived metabolites (including lignin and flavonoids) can compete for carbon resources and consequently restrict starch deposition, as reported in cassava and sweet potato [50,51]; meanwhile, in stem-derived potato tubers, sucrose not only serves as a carbon substrate but also acts as a signaling molecule that, together with R2R3-MYB transcription factors and StmiR858, regulates flavonol biosynthesis [52]. These suggest partial partitioning of endogenous carbon skeletons between primary storage metabolism and flavonoid secondary metabolism in tubers of *S. grosvenorii*. Nevertheless, an in vitro tissue culture system with sufficient exogenous carbon supply was adopted. The overall carbon reserve could simultaneously meet the dual material requirements for starch storage and flavonoid biosynthesis. Carbon trade-offs between starch and phenylpropanoid pathways, together with sucrose-mediated transcriptional regulation, determine the flux toward flavonoid backbone formation. Nevertheless, de novo-synthesized flavonoid aglycones are chemically unstable in plants and require post-translational modification for stable storage and biological function [53,54]. Glycosylation modification improves the stability and biological activity of flavonoids, facilitating the accumulation of functional defensive metabolites in plants [55,56]. Accordingly, abundant glycosylated flavonoids may strengthen stress, insect and disease resistance of in vitro-cultured tubers, while accumulated starch provides energy substrates for subsequent tuber sprouting.

MYB, bHLH and ERF subfamilies are master transcription factor families governing storage organ morphogenesis, carbohydrate metabolic reprogramming and flavonoid biosynthesis in tuber crops, and numerous functional studies have documented their biological functions [30,52,57,58]. The correlation heatmap showed that several candidate regulators, such as *SgbHLH093*, *SgMYB306*, *SgERFC3*, *SgRAP2-7* and *SgRAP2-4*, were linked to metabolites involved in starch and sucrose metabolism and flavonoid biosynthesis during in vitro tuberization of *S. grosvenorii*. The *bHLH093*, *Myb3* and *RAP2-7* have been previously described in other plant species, indicating the conservation of these regulatory components [31,32,33]. We have also selected *SPS4*, *GBSS1* and some of them for qRT-PCR validation (Figure S1A). *SgUBQ* was selected as the reference gene for qRT-PCR normalization [59,60] and exhibited relatively stable expression across all sampled developmental stages of tuber induction in our qRT-PCR samples. Expression trends detected by qRT-PCR were highly consistent with those obtained from the transcriptome dataset, with a Pearson correlation coefficient of 0.871 (*p* = 5.03 × 10^−12^), supporting the credibility of our transcriptome dataset. *SgRAP2-4* showed a slight discrepancy between the two detection methods (Figure S1B,C). This minor divergence may result from subtle physiological differences among in vitro cultured samples and different quantitative normalization approaches of the two techniques, without affecting the overall validity of our transcriptome analysis. Interestingly, qRT-PCR revealed downregulation of *SgRAP2-4* and *SgERFC3* (Figure S1A). These TFs showed both positive and negative correlations with key metabolites (Figure 6C), suggesting that TFs may differentially modulate distinct branches of metabolic networks.

*S. grosvenorii* is a typical medicinal and edible plant, and mogrosides represent its core bioactive constituents. Based on the known biosynthetic background of mogrosides, we performed targeted screening to identify genes involved in this pathway. We retrieved several previously reported genes for mogroside biosynthesis from our transcriptome dataset, including squalene epoxidase (*SgSQE*), cucurbitadienol synthase (*SgCDS*), cytochrome P450 87D18 (*SgCYP87D18*), and uridine diphosphate glycosyltransferase 74AC1 (*SgUGT74AC1*) (Figure S2) [4,61]. SgCDS catalyzes the formation of cucurbitadienol, a reaction that constitutes the first rate-limiting step in the biosynthesis of the mogroside backbone [62]. SgUGT74AC1 mediates the glycosylation of mogrol to produce mogroside IE [63,64]. *SgSQE* and *SgUGT74AC1* members exhibited complex expression patterns. By contrast, *SgCDS* and *SgCYP87D18* showed relatively high expression levels at 0 d and 90 d, with decreased transcript abundance during the middle stage of culture. Unfortunately, a series of intermediate metabolites in the mogroside biosynthetic pathway were not identified in our widely targeted metabolomics data. These indicate that the genetic program governing mogroside biosynthesis may be activated at the molecular level. The accumulation of intermediate metabolites and final products may require a longer certain developmental timeframe. Extending the culture period in future experiments will likely enable the detection of precursors such as cucurbitadienol and mogrol. These findings support the potential of *S. grosvenorii* tubers as a platform for producing mogrosides precursors, given that mogrosides are high-value natural sweeteners for the food industry.

There are also some limitations in our research. First, all samples used in this study were obtained from in vitro tissue culture. The axenic culture system, exogenous supplementation of plant growth regulators, and fixed nutrient composition of artificial medium differ substantially from the natural field cultivation conditions. Second, integrative analysis of the transcriptome and widely targeted metabolome only reveals statistical correlations between genes and metabolites, rather than direct causal regulatory relationships between transcription factors and metabolite changes. The putative regulatory roles of MYB, bHLH and ERF transcription factors were inferred from multi-omics association data and qRT-PCR quantification, lacking direct molecular evidence from yeast one-hybrid assay, dual-luciferase reporter assay, and gene overexpression or silencing assays. Although these transcriptional and metabolic shifts are temporally coupled with in vitro tuber formation of *S. grosvenorii*, we cannot conclude that these molecular events directly drive tuber development. Establishing a direct functional link requires further gene manipulation experiments. Subsequent research will focus on systematic in vivo and in vitro functional characterization of core candidate transcription factor genes to elucidate their molecular mechanisms in targeting downstream structural genes and mediating tuber expansion as well as carbon partitioning. Meanwhile, tubers derived from in vitro culture should be transplanted into field conditions to compare their growth rate and disease and pest resistance with conventionally propagated cuttings. Benefiting from the accumulation of flavonoids, starch, and other metabolites, plants regenerated from these tubers may also produce fruits with improved quality and flavor. In addition, further in-depth research will be conducted on the markedly upregulated genes involved in mogroside biosynthesis at the late developmental stage to dissect the regulatory pathway governing the temporal accumulation of mogrosides, thereby supporting the utilization of *S. grosvenorii* as a natural sweetener resource.

## 5. Conclusions

This study systematically characterized the dynamic profiles of in vitro-induced tuber formation of *S. grosvenorii* at seven consecutive developmental stages. The results revealed that the in vitro-induced tuber formation of *S. grosvenorii* was accompanied by the synthesis of defensive secondary metabolites, including flavonoids, at the early developmental stage to resist in vitro stresses, while substantial carbon flux was diverted to the biosynthesis of storage substances such as sucrose and starch. Candidate MYB, bHLH and ERF transcription factors, such as *SgbHLH093* and *SgRAP2-4*, may putatively modulate starch and sucrose metabolism and flavonoid biosynthesis during tuber development.

Conclusively, this study preliminarily constructed a metabolic reprogramming framework underlying the morphogenesis of in vitro tubers of *S. grosvenorii*. The outcomes provide multi-omics evidence for exploring the developmental mechanisms of storage organs in medicinal cucurbit plants and lay a solid theoretical foundation for yield improvement and targeted selective breeding of active ingredients—particularly mogrosides as natural sweeteners and functional food constituents—in *S. grosvenorii*.

## Data Availability

The RNA-seq raw sequencing data generated in this study have been deposited in the NCBI Sequence Read Archive (SRA) under accession number PRJNA1521120. All other data supporting the conclusions of this study are included within the article and its Supplementary Materials.

## Supplementary Materials

The following supporting information can be downloaded at https://www.mdpi.com/article/10.3390/plants15213319/s1. Figure S1: qRT-PCR validation and comparison with transcriptome data. Figure S2: Expression of *SgSQE*, *SgCDS*, *SgCYP87D18* and *SgUGT74AC1* genes. Table S1: Ordinary one-way ANOVA and multiple comparisons. Table S2: The primers used for qRT-PCR. Table S3: OPLS-DA model and permutation test parameters. Table S4: All DEG summary. Table S5: All sample data of metabolites; Table S6: sigMetabolites summary. Table S7: DEGs of the starch and sucrose metabolism. Table S8: DEGs of the flavonoid biosynthesis pathway. Table S9: TFs from WGCNA module–brown–turquoise–pink; Table S10: Starch and soluble sugar content.
